# Inguinal and scrotal extramammary Paget’s disease: ^18^F-FDG PET/CT imaging

**DOI:** 10.1590/0100-3984.2016.0144

**Published:** 2018

**Authors:** Kelly Tung, Ba D. Nguyen

**Affiliations:** 1 Department of Radiology, Mayo Clinic, Arizona, Scottsdale, AZ, USA.

*Dear Editor,*

An 87-year-old man presented to our institution for investigation of an intertriginous rash, involving the left inner thigh, scrotum, and perineum, which had been neglected for a few years. The lesion was diagnosed as extramammary Paget’s disease (EMPD). Due to the potential for EMPD to be associated with gastrointestinal and genitourinary malignancies, a thorough clinical and imaging evaluation was performed, the results of which were negative. The patient opted for symptomatic care only. Two years later, he returned to our institution with soft tissue swelling and edema of the left lower extremity, scrotum, and penis, with a nodular scrotal lesion and bilateral inguinal adenopathy (Figure 1). Positron emission tomography/computed tomography (PET/CT) showed ^18^F-fluorodeoxyglucose (FDG)-avid lesions of the left scrotum, left inguinal lymph node, left pelvic lymph node, T12 vertebra, ribs, and left scapula (Figure 2). Biopsy of the left inguinal adenopathy showed signet-ring cells. Radiation therapy was initiated, resulting in partial improvement, followed by chemotherapy with carboplatin and paclitaxel. Unfortunately, the patient died, due to disease progression, at five months after the ^18^F-FDG PET/CT imaging.

**Figure 1:**
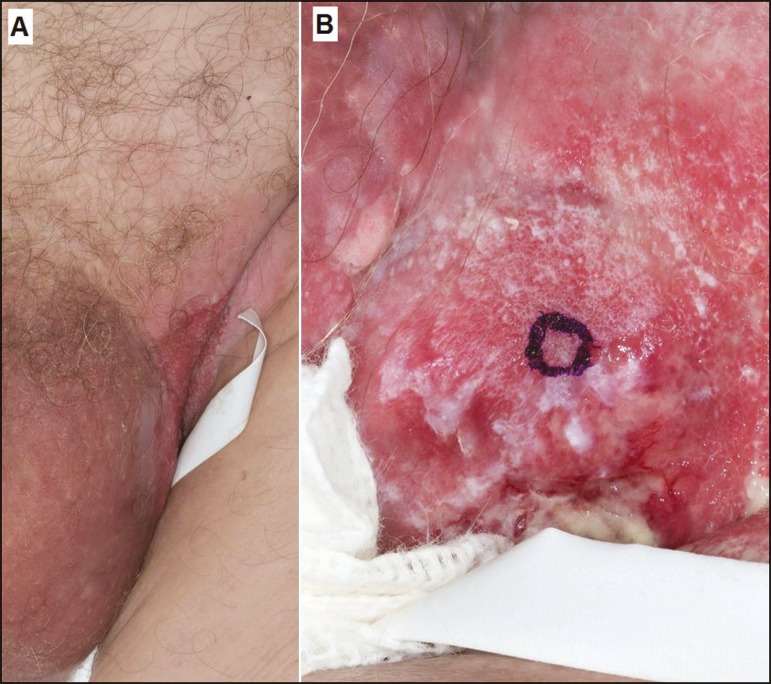
Photographs of a patient with EMPD. **A:** Lesions of the scrotum and left inguinal region. **B:** Close-up of the left inguinal lesion with a beefy red center and macerated whitish border.

**Figure 2:**
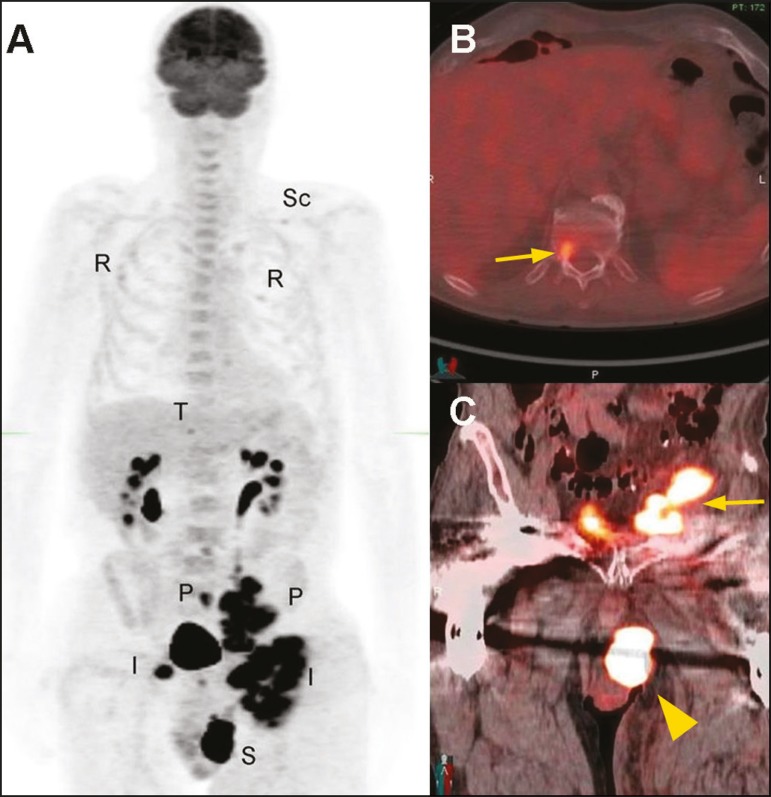
Maximum-intensity projection ^18^F-FDG PET/CT (**A**) showing the left scrotal lesion (S), bilateral inguinal adenopathy (I), and pelvic adenopathy (P), as well as osseous metastases involving the T12 vertebra (T), multiple ribs (R), and the left scapula (Sc). Fused axial and coronal ^18^F-FDG PET/CT images showing the T12 vertebra metastasis (arrow in **B**), together with the left scrotal lesion and left pelvic lymph node metastasis (arrowhead and arrow, respectively, in **C**).

EMPD is a rare intraepithelial adenocarcinoma that typically gives rise to a pruritic rash at sites with numerous apocrine glands, such as the perineum, axilla, eyelids, scalp, and buttocks^(^^1^^)^. The disease occurs predominantly in patients over 50 years of age. In the Caucasian population, females are more affected than are males, whereas there is a predominance of males among EMPD patients in the Asian population^(^^2^^)^. The diagnosis of EMPD is based on the identification of Paget’s cells with prominent nuclei and abundant lightly stained cytoplasm on hematoxylin-eosin staining^(^^1^^,^^3^^)^. The disease can arise from two major pathological mechanisms^(^^4^^)^: as an *in situ* intraepithelial adenocarcinoma which has the potential for local invasion and subsequent metastasis; and as pagetoid spread of a visceral malignancy. Isolated EMPD, without a coexisting internal primary lesion, is usually an indolent, slow-growing cancer that rarely metastasizes. Rare invasive EMPD has a propensity to metastasize to inguinal nodal basins^(^^5^^)^. EMPD involving the external genitalia has a strong association with gastrointestinal and genitourinary adenocarcinomas^(^^4^^,^^6^^)^. A small subset of invasive EMPD cases show signet-ring cell morphology with extracellular mucin. Immunohistochemical analysis establishes the distinction between signet-ring cells intrinsic to EMPD and those originating from coexisting visceral neoplasms^(^^7^^,^^8^^)^. Poor prognostic factors include dermal invasion, nodular skin lesions, lymph node involvement, and distant metastasis^(^^3^^)^. Given the multiple presentations of EMPD and their varying prognoses, there is a need to identify distant metastases and the primary visceral tumor: that effort is facilitated by functional ^18^F-FDG PET/CT imaging^(^^1^^-^^6^^)^.

## References

[1] Zhu Y, Ye DW, Yao XD (2009). Clinicopathological characteristics, management and outcome of
metastatic penoscrotal extramammary Paget's disease. Br J Dermatol.

[2] Kim JC, Kim HC, Jeong CS (1999). Extramammary Paget's disease with aggressive behavior: a report
of two cases. J Korean Med Sci.

[3] Li ZG, Qin XJ (2015). Extensive invasive extramammary Paget disease evaluated by F-18
FDG PET/CT: a case report. Medicine (Baltimore).

[4] Cho SB, Yun M, Lee MG (2005). Variable patterns of positron emission tomography in the
assessment of patients with extramammary Paget's disease. J Am Acad Dermatol.

[5] Aoyagi S, Sato-Matsumura KC, Shimizu H (2005). Staging and assessment of lymph node involvement by
18F-fluorodeoxyglucose-positron emission tomography in invasive extramammary
Paget's disease. Dermatol Surg.

[6] Niederkohr RD, Gambhir SS (2006). F-18 FDG PET/CT imaging of extramammary Paget disease of the
perianal region. Clin Nucl Med.

[7] Shu B, Shen XX, Chen P (2016). Primary invasive extramammary Paget disease on penoscrotum: a
clinicopathological analysis of 41 cases. Hum Pathol.

[8] Uchimiya H, Yonekura K, Hashiguchi T (2009). Extramammary Paget's disease with prominent signet-ring
cells. J Dermatol.

